# Genome-wide identification, gene cloning, subcellular location and expression analysis of SPL gene family in *P. granatum* L

**DOI:** 10.1186/s12870-021-03171-7

**Published:** 2021-08-28

**Authors:** Bianbian Li, Yujie Zhao, Sha Wang, Xinhui Zhang, Yongwei Wang, Yu Shen, Zhaohe Yuan

**Affiliations:** 1 Co-Innovation Center for Sustainable Forestry in Southern China, Nanjing Forestry University, Nanjing, 210037 China; 2 College of Forestry, Nanjing Forestry University, Nanjing, 210037 China

**Keywords:** Pomegranate, SPL gene family, Gene cloning, Subcellular localization, Gene expression

## Abstract

**Backgrounds:**

Pomegranate is an excellent tree species with nutritional, medicinal, ornamental and ecological values. Studies have confirmed that SPL factors play an important role in floral transition and flower development.

**Results:**

Used bioinformatics methods, 15 SPL (SQUAMOSA promoter-binding protein-like) genes were identified and analyzed from the ‘Taishanhong’ pomegranate (*P. granatum* L.) genome. Phylogenetic analysis showed that PgSPLs were divided into six subfamilies (G1 ~ G6). PgSPL promoter sequences contained multiple *cis*-acting elements associated with abiotic stress or hormonal response. Based on the transcriptome data, expression profiles of different tissues and different developmental stages showed that *PgSPL* genes had distinct temporal and spatial expression characteristics. The expression analysis of miR156 in small RNA sequencing results showed that miR156 negatively regulated the expression of target genes. qRT-PCR analysis showed that the expression levels of *PgSPL2*, *PgSPL3*, *PgSPL6*, *PgSPL11* and *PgSPL14* in leaves were significantly higher than those in buds and stems (*p* < 0.05). The expression levels of *PgSPL5*, *PgSPL12* and *PgSPL13* in flower buds were significantly higher than that in leaves and stems (*p* < 0.05). The full-length of coding sequence of *PgSPL5* and *PgSPL13* were obtained by homologous cloning technology. The full length of *PgSPL5* is 1020 bp, and *PgSPL13* is 489 bp, which encodes 339 and 162 amino acids, respectively. Further investigation revealed that *PgSPL5* and *PgSPL13* proteins were located in the nucleus. Exogenous plant growth regulator induction experiments showed that *PgSPL5* was up-regulated in leaves and stems. *PgSPL13* was up-regulated in leaves and down-regulated in stems. When sprayed with 6-BA, IBA and PP333 respectively, *PgSPL5* and *PgSPL13* were up-regulated most significantly at P2 (bud vertical diameter was 5.1 ~ 12.0 mm) stage of bisexual and functional male flowers.

**Conclusions:**

Our findings suggested that *PgSPL2*, *PgSPL3*, *PgSPL6*, *PgSPL11* and *PgSPL14* played roles in leaves development of pomegranate. *PgSPL5*, *PgSPL12* and *PgSPL13* played roles in pomegranate flower development. *PgSPL5* and *PgSPL13* were involved in the response process of different plant hormone signal transduction in pomegranate development. This study provided a robust basis for further functional analyses of *SPL* genes in pomegranate.

**Supplementary Information:**

The online version contains supplementary material available at 10.1186/s12870-021-03171-7.

## Background

Transcription factors can specifically bind with *cis*-acting elements of the promoter, which activate or suppress the expression of downstream target genes. The transcription factors involved in floral organ development mainly include MADS-box [1], bHLH [2], YABBY [3] and SPL [4]. The plant-specific SPL (SQUAMOSA promoter binding protein-like) transcription factor is involved in the formation and development of flowers, leaf morphogenesis, and other biological processes [5, 6]. It is known that SPL family members have a highly conserved SBP domain consisted of 79 amino acids, and have two typical zinc finger structures including C3H (C-C-CH) and C2HC (C-C-H-C) [7]. The C-terminal of the SPL proteins combine with the second zinc finger promoting its combination with the DNA to perform function [8].

SPL family genes were originally isolated from *A. majus* [9]. They regulate early flowering of plants by binding with the SQUAMOSA promoter of MADS-box family genes [9]. In *Arabidopsis*, 17 *SPL* genes have been isolated and named as *SPL1* ~ *SPL16*. *AtSPL13* are a pair of paralogous genes, which are named as *SPL13A* and *SPL13B* [10]. In recent years, many researchers have successively identified SPL family genes in other species, such as 28 *SPL* genes in poplar (*Populus trichocarpa*) [11], 15 in tomato (*Solanum lycopersicum*) [12], 19 in rice (*O. sativa*) [13], 27 in apple (*M. domestica* Borkh.) [14] and 18 *SPL* genes in grape (*Vitis vinifera* L.) [15].

MicroRNAs (miRNAs) are a class of endogenous non-coding RNAs, about 20–24 nt in length, that are derived from primary miRNA transcripts (pri-miRNAs) containing a stem-loop secondary structure [16, 17]. They are known to suppress the expression of target genes at the post-transcriptional level via mRNA cleavage or translational inhibition [18, 19]. In plants, miRNAs act crucial roles in plant organ development [20], stress tolerance [21], phytohormone signaling [22], growth phase change [23], and disease resistance [21]. miR156 is a highly conserved class of miRNAs, which involves in regulating the transformation of plants from vegetative stage to reproductive stage [24–26]. Among 17 *SPL* genes in *Arabidopsis*, 11 members have the miR156 binding sites [27–30]. According to the size of the SBP domain, the *SPL* genes regulated by miR156 can be divided into two categories. One includes *SPL3*, *SPL4*, and *SPL5*, which encode smaller proteins and promote flower formation; the other of *SPL9* and *SPL15* encode larger proteins, which mainly promote leaf formation and flowering [31, 32].

Previous studies found that *SPL2* regulates floral organ development and plant fertility by activating *ASYMMETRIC LEAVES 2* (*AS2*) [33]. *SPL3/4/5* can activate the expression of *LEAFY* (*LFY*), *FRUITFULL* (*FUL*) and *APETALA1* (*AP1*) in floral meristems [34, 35]. As a gene not regulated by miR156, *SPL8* plays a redundant role with other miR156-targeted *SPL* genes, which can regulate the development of microspore sac, megasporogenesis, hairy body formation on sepals, and stamen filament elongation, so as to control the male fertility of plants [36]. *SPL9* and *SPL10* control flowering time by directly activating miR172 [28]. *SPL15* is a positive regulator for flowering stage, and it cooperates with *SUPPRESSOR OF CONSTANS 1* (*SOC1*) to induce *Arabidopsis* flower development [37]. *SPL2*, *SPL8*, *SPL9* and *SPL15* have the effect of maintaining male fertility in *Arabidopsis* [38, 39].

Pomegranate is an excellent tree species with nutritional, medicinal, ornamental and ecological values [40]. Studies have confirmed that SPL factors play an important role in floral transition and flower development [32, 34, 35, 37, 41]. Whether it plays a role in pomegranate development has not been reported. Based on the whole genome data of ‘Taishanhong’ pomegranate, the members of SPL family were identified by bioinformatics methods. Their physical and chemical characteristics were analyzed. The evolution relationship, gene structure, conserved motifs, difference of *cis*-acting elements and expression of gene tissue characteristics of each member were explored. The target sites of miR156 were predicted. The temporal and spatial expression characteristics of *PgSPL5* and *PgSPL13* genes under the treatment of cloning, subcellular localization and growth regulator were analyzed to provide reference for revealing their biological functions in pomegranate flower induction.

## Results

### Identification and characterization of SPL transcription factors

Fifteen SPL gene family members were identified from the genome of ‘Taishanhong’ pomegranate. For the convenience of subsequent analysis, they were named as *PgSPL1* ~ *PgSPL15* (Table 1). The protein sequence and physicochemical properties of PgSPL members were analyzed. The results showed that the length of *PgSPL* genes changed greatly. The shortest protein encoded 162 amino acids (*PgSPL13*), the longest is 1049 amino acids (*PgSPL9*), and the molecular weight of the protein ranged from 18,563.02 kDa to 115,942.26 kDa. The theoretical isoelectric point ranged from 5.38 to 9.39, with *PgSPL13* as low as 5.38 and *PgSPL14* as high as 9.39. It contains 10 acidic proteins and 5 basic proteins. These results provided a theoretical basis for further purification, activity and function research of PgSPL proteins. The results of subcellular location showed that members were expressed in the nucleus, except *PgSPL4*, *PgSPL6* and *PgSPL12* in the nucleus and cytoplasm.

**Table 1:** Member characteristics of SPL gene family in pomegranate

Gene Name	Gene ID	Location	Length	pI	Mw(kDa)	Group	Subcellular Localization
*PgSPL1*	*Pg000744.1*	scaffold1:4774751:4776860	500	6.37	54,138.16	G3	Nuclear
*PgSPL2*	*Pg003831.1*	scaffold12:3285330:3286080	196	9.16	21,938.35	G5	Nuclear
*PgSPL3*	*Pg005590.1*	scaffold13:3198475:3200620	527	8.87	57,576.09	G3	Nuclear
*PgSPL4*	*Pg008456.1*	scaffold16:1476693:1481665	778	5.53	86,532.31	G1	Cytoplasm/Nucleur
*PgSPL5*	*Pg014414.1*	scaffold25:1722378:1724406	339	8.89	36,911.79	G4	Nuclear
*PgSPL6*	*Pg016042.1*	scaffold3:3668488:3670385	389	8.87	41,945.73	G2	Cytoplasm/Nucleur
*PgSPL7*	*Pg016049.1*	scaffold3:3581401:3584942	493	8.4	54,118.39	G3	Nuclear
*PgSPL8*	*Pg017676.1*	scaffold33:999708:1004761	999	6.76	111,303.95	G6	Nuclear
*PgSPL9*	*Pg019080.1*	scaffold39:1554086:1558442	1049	8.06	115,942.26	G6	Nuclear
*PgSPL10*	*Pg020599.1*	scaffold42:1057229:1061842	986	5.92	109,247.27	G6	Nuclear
*PgSPL11*	*Pg027304.1*	scaffold72:239136:241212	391	8.61	42,020.97	G2	Nuclear
*PgSPL12*	*Pg028546.1*	scaffold80:561916:564888	402	9.4	42,499.1	G3	Cytoplasm/Nucleur
*PgSPL13*	*Pg029123.1*	scaffold84:570880:572840	162	5.38	18,563.02	G5	Nuclear
*PgSPL14*	*Pg029352.1*	scaffold87:784733:785930	171	9.39	19,317.66	G5	Nuclear
*PgSPL15*	*Pg030861.1*	scaffold99:46274:51496	334	8.9	36,242.96	G3	Nuclear

*Mw* molecular weight, *pI* isoelectric points

### Phylogenetic analysis

**Fig. 1:**
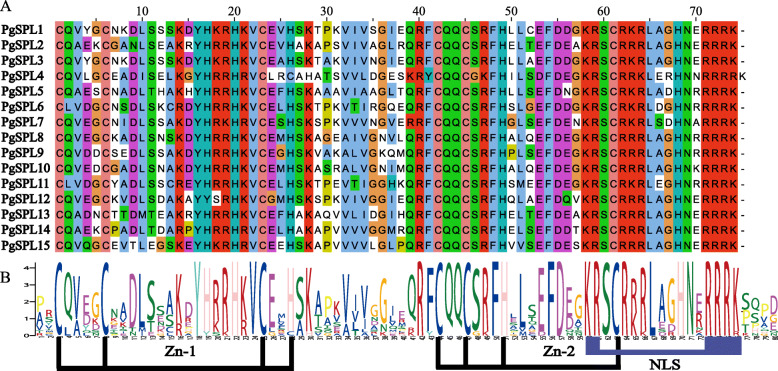
Multiple alignment of the SBP domains of the pomegranate SPL proteins. **a** Multiple alignment of the SBP domains of the PgSPL proteins was obtained with DNAMAN software. The two conserved zinc finger structures (Zn-1, Zn-2) and NLS are shown. **b** Sequence logo of the SBP domain of PgSPL proteins. The height of the letters within each stack represents the relative frequency of the corresponding amino acids

Multiple sequence alignment results (Fig. 1) showed that each SPL transcription factor family member contained a highly conserved SBP domain composed of 79 amino acid residues. They all contain two zinc finger structures Cys-Cys-Cys-His (C3H), Cys-Cys-His-Cys (C2HC) and a nuclear localization signal (NLS). The N-terminal zinc finger structure of *PgSPL4* is Cys4 (C4), which is different from other members.

**Fig. 2:**
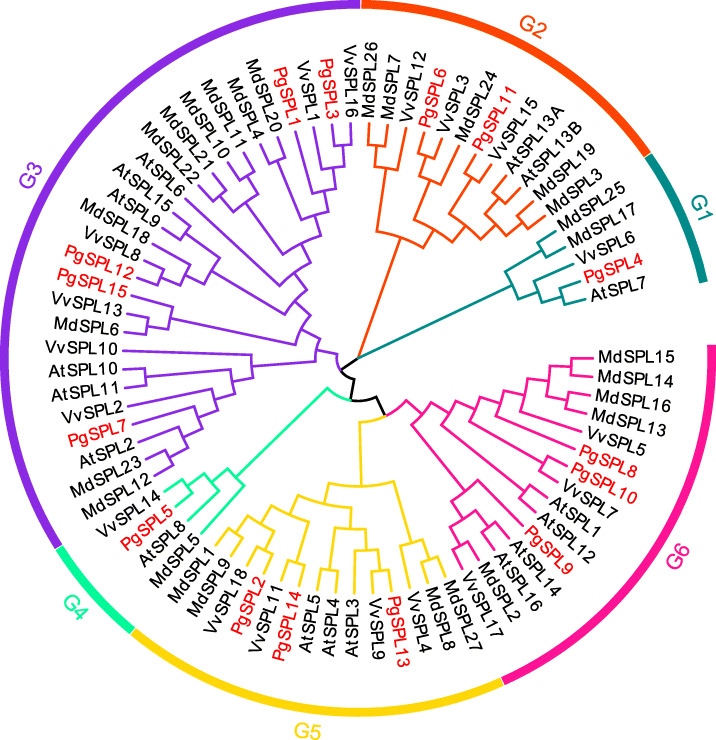
Phylogenetic tree of four species: *Punica granatum*, *Arabidopsis*, *M. domestica* and *Vitis vinifera*

The phylogenetic tree results showed that 15 *SPL* genes of pomegranate were clustered into six subfamilies (G1 ~ G6) (Fig. 2) [42, 43]. Among these six subgroups, G1 subgroup has a distant genetic relationship with other subgroups, forming a relatively independent branch. Further analysis found that the N-terminal zinc finger structure in the SBP domain of the five members of the G1 subgroup *AtSPL7*, *PgSPL4*, *VvSPL6*, *MdSPL17*, and *MdSPL25* is C4, while that of all members in the other subgroups is C3H. The members of SPL transcription factor family in G3 subgroup were the most widely distributed. Among them, 5 of 15 pomegranate *SPL* genes were distributed in G3 subgroup. G4 subgroup each has a pomegranate, *Arabidopsis*, apple and grape SPL gene. There were two *PgSPL* genes in G2 subgroup, and three *PgSPL* genes in G5 and G6.

### Gene structure analysis and motif identification of *PgSPLs*

**Fig. 3:**
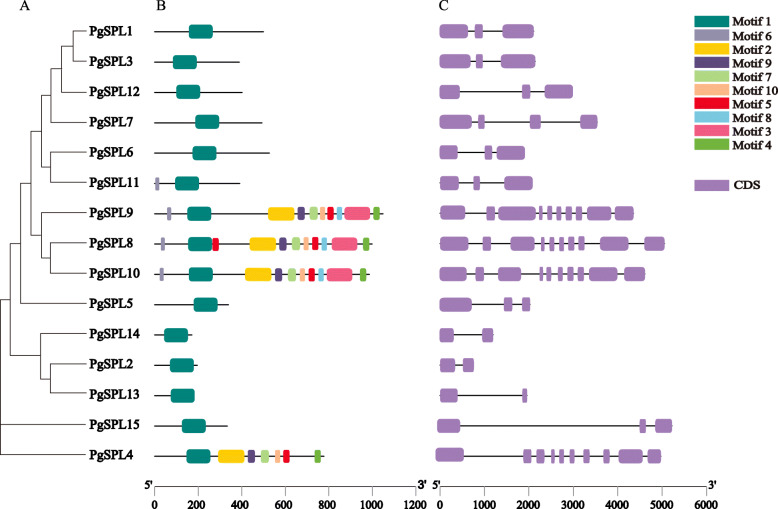
Phylogenetic tree, conserved motifs and gene structures of the PgSPL gene family. **a** The phylogenetic tree was constructed based on the full-length sequences of PgSPL proteins. **b** The architecture of conserved protein motifs was indicated by different colored blocks. **c** The exon-intron structure of *PgSPL* genes

The results of *PgSPLs* gene structure showed that the number of intron was 1 ~ 9 (Fig. 3c). The members of G1 and G6 contained nine introns. In the G2, G3 and G4 subgroups, the members had 2 introns except *PgSPL7* possessed 3 introns. PgSPLs in G5 subgroup contained only one intron.

Ten conserved motifs of PgSPLs were identified (Fig. 3b). All PgSPL proteins contained Motif1, the SBP domain, which is composed of about 79 amino acids. PgSPLs members in the same subfamily showed similar gene structure and protein conservation motif. In G2 ~ G5 subgroups, all members only contained the SBP domain, except *PgSPL11*. However, members in G1 and G6 subgroups have some no or atypical domains in other subgroups, the differential distribution of these conserved motifs might be the reasons for gene functional difference.

### *Cis*-acting elements located in *SPL* gene promoters

**Fig. 4:**
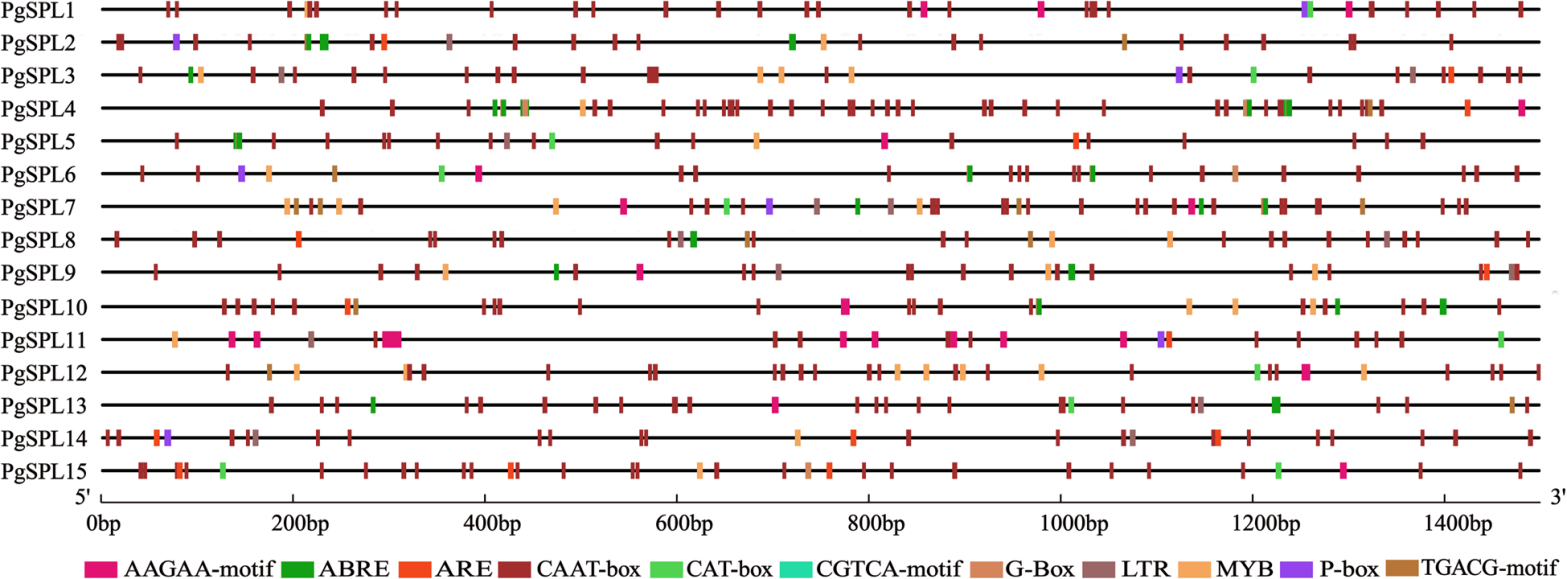
Cis-elements analysis of pomegranate *SPL* genes

The *cis*-acting elements contained a wide variety, ranging from 23 in *PgSPL14* to 42 in *PgSPL7* (Fig. 4). PgSPLs respond to different plant hormones and abiotic stress signals, including AAGAA-motif and ABRE, the *cis*-acting elements involved in abscisic acid response, gibberellin response element P-box, methyl jasmonate response element CGTCA-motif and TGACG-motif, the necessary *cis*-regulatory element ARE for anaerobic induction, the *cis*-acting regulatory element CAT-box which is related to meristem expression, the *cis*-acting regulatory element G-Box which is involved in photoreactivity, the cold stress response element LTR, the drought-induced response element MBS, and the common *cis*-acting element CAAT-box in the promoter and enhancer regions. Among 15 *PgSPLs*, 11 genes contained AAGAA-motif and ABRE elements, 7 genes contained P-box elements, and 8 contained CGTCA-motif and TGACG-motif elements. Ten members contained ARE elements, 9 members contained CAT-box elements, 5 genes contained G-Box elements, 9 genes contained stress-related elements LTR and MBS, and all *PgSPL* genes contained enhancer elements CAAT-box.

### Prediction of *PgSPL* genes targeted by miR156

**Fig. 5:**
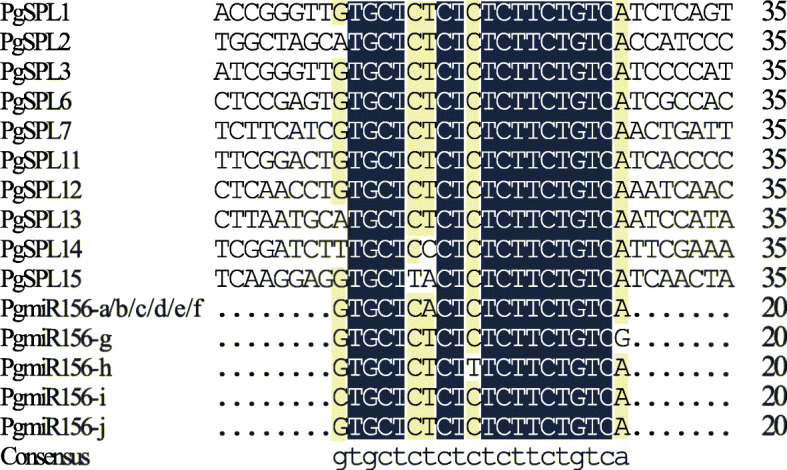
Mutiple alignment of miR156 complementary sequences with *PgSPL* genes

Many reserches have shown that most *SPL* genes are regulated by miR156, and miR156 target sites are located in the coding region or 3’UTR region. In order to determine post-transcriptional regulatory mechanism in pomegranate, miR156 target sites were searched at the coding regions and 3’UTR regions of *PgSPLs*. The results showed that *PgSPL1*, *PgSPL2*, *PgSPL3*, *PgSPL6*, *PgSPL7*, *PgSPL11*, *PgSPL12*, *PgSPL13*, *PgSPL14* and *PgSPL15* were potential targets for miR156, and these genes were clustered in G2, G3 and G5 subgroups. The *PgSPLs* with potential miR156 targets in the coding region belong to G2 and G3, while *PgSPL2*, *PgSPL13* and *PgSPL14* with targets sites in 3’UTR belong to the G5 subgroup. *PgSPL* gene sequences were compared with the mature *PgmiR156* sequence, and the results showed that more than half of PgSPL contained sequences complementary to *PgmiR156* sequence with no more than two mismatched bases (Fig. 5). This result is similar to other species [43, 44].

### Expression patterns by transcriptome data

**Fig. 6:**
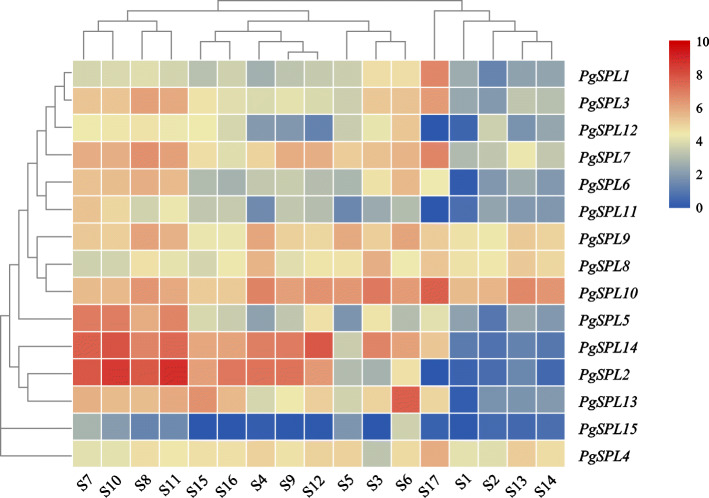
The heatmap of expression of *SPL* genes in different tissues of pomegranate. S1: Outer seed coat; S2: Inner seed coat; S3: Pericarp; S4; Flower; S5: Root; S6: Flesh leaf; S7: Bisexual flowers (3.0 ~ 5.0 mm); S8: Bisexual flowers (5.1 ~ 13.0 mm); S9: Bisexual flowers (13.1 ~ 25.0 mm); S10: Functional male flowers (3.0 ~ 5.0 mm); S11: Functional male flowers (5.1 ~ 13.0 mm); S12: Functional male flowers (13.1 ~ 25.0 mm); S13: Inner seed coat of ‘Tunisia’; S14: Inner seed coat of ‘Baiyushizi’; S15: Mix of leaves, flowers, fruit and roots of ‘Black127’; S16: Mix of leaves, flowers, fruit and roots of ‘nana’; S17: Peels of ‘Wonderful’ (cultivars S1 ~ S6 are ‘Dabenzi’, cultivars S7 ~ S13 are ‘Tunisia’)

As shown in Fig. 6, comparative analysis of the transcriptome was in different tissues. The results showed that the expression of *PgSPL1* and *PgSPL3* were high in pericarp, and were highly expressed in flowers, more than those in seed coat. *PgSPL2* expression was high in flowers, moderate in inner and outer seed coat, but not in ‘Wonderful’ pericarp. There was no significant difference in *PgSPL4* expression levels in all tissues. *PgSPL5* showed high expression level in the stages of bisexual flowers I (3.0 ~ 5.0 mm), functional male flowers I (3.0 ~ 5.0 mm) and II (5.1 ~ 13.0 mm), and lower in inner seed coat. *PgSPL6* is expressed at a low level in the outer seed coat. *PgSPL7* showed higher expression in the outer seed coat and bisexual flowers II (5.1 ~ 13.0 mm). *PgSPL8* and *PgSPL9* exhibited similar patterns of expression. *PgSPL10* was highly expressed in ‘Wonderful’ pericarp. *PgSPL11* and *PgSPL12* were not expressed in ‘Wonderful’ pericarp. *PgSPL13* was expressed at the highest level in leaves, maintained at a high level during the stage I and II of bisexual flowers and functional male flowers, but low expression in outer seed coat. *PgSPL14* had the highest expression levels of functional male flowers I and III. The highest level of *PgSPL15* was found in leaves, while the expression was low or not expressed in other tissues.

**Fig. 7:**
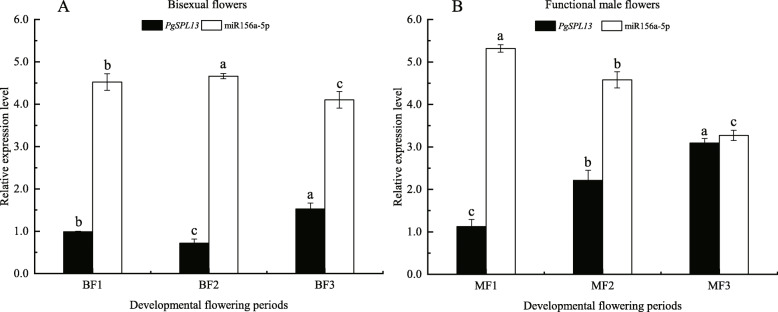
Relative expression of miR156a-5p and *PgSPL13* genes at the development of Pomegranate flowers. **a** Relative expression of miR156a-5p and *PgSPL13* genes at bisexual flowers. BF1, BF2, BF3 correspond to three developmental stages (bud vertical diameter were 3.0 ~ 5.0 mm, 5.1 ~ 13.0 mm, 13.0 ~ 20.0 mm) of Bisexual flowers, respectively. **b** Relative expression of miR156a-5p and *PgSPL13* genes at functional male flowers. MF1, MF 2, MF 3 correspond to three developmental stages (bud vertical diameter were 3.0 ~ 5.0 mm, 5.1 ~ 13.0 mm, 13.0 ~ 20.0 mm) of Functional male flowers. The vertical bar in bar graph is standard error. Bars with different letters within each panel are significant differences at *p* < 0.05 according to the Turkey’s test

We also validated *miR156a-5p* and *PgSPL13* that have differentially expression patterns during the different stages of pomegranate flowers development (Fig. 7). It was found that during the development of bisexual flowers (Fig. 7a), the expression of *miR156a-5p* first increased and then decreased, while the expression of *PgSPL13* first decreased and then increased. And the expression was significantly different in each period (*p* < 0.05). During the development of functional male flowers (Fig. 7b), the expression of *miR156a-5p* was significantly decreased, while the expression of *PgSPL13* was significantly increased (*p* < 0.05).

### qRT-PCR analysis of *PgSPLs*

**Fig. 8:**
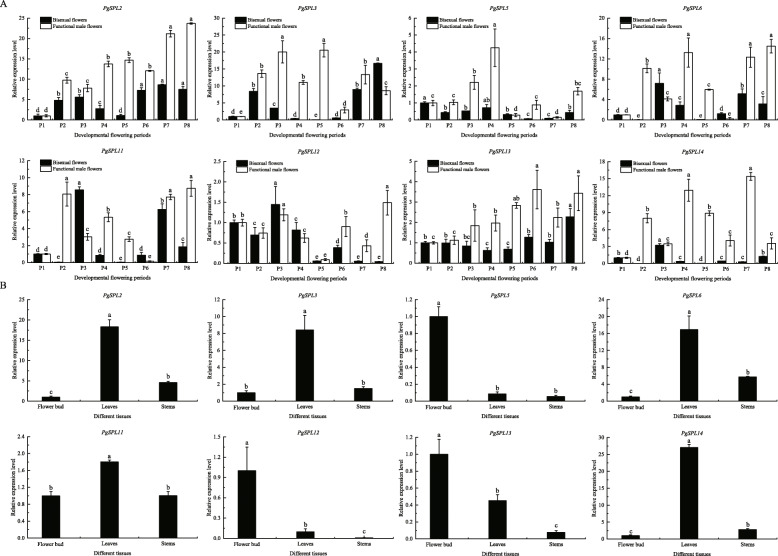
Expression of *PgSPLs* in different tissues of pomegranate. Flower buds, leaves and stems are all young tissues of pomegranate. The vertical bar in bar graph is standard error. Bars with different letters within each panel are significant differences at *p* < 0.05 according to the Turkey’s test

From Fig. 6, eight differentially expressed *PgSPLs* genes were screened out, the expression patterns of *PgSPL* genes in different tissues of pomegranate was analyzed by qRT-PCR. The results are shown in Fig. 8, the expression levels of *PgSPL2* were the highest at P7 and P8 stages of two types of flowers, and the expression levels at P5 stage of the bisexual flowers were significantly lower than those at other stages (*p* < 0.05). We found that the expression levels of *PgSPL3* in P4 ~ P6 stages of bisexual flowers were significantly lower than those in other stages. It was significantly increased in P6 ~ P8 stages (*p* < 0.05), and it was higher in P2 ~ P5 stages of functional male flowers. *PgSPL5* had higher expression in functional male flowers than in bisexual flowers, and its expression level showed a trend of up-down-up during the development of bisexual flowers. At P2 ~ P4 stages of functional male flowers, the expression levels of *PgSPL5* were significantly increased (*p* < 0.05), and reached the highest level at the P4 stage, then slightly downregulated. The expression levels of *PgSPL6* in the P2 and P5 stages of bisexual flowers were significantly lower than those in other stages (*p* < 0.05). The expression levels of *PgSPL6* in the P8 stage of functional male flowers were the highest, followed by those in the P4 and P7 stages, and then the P6 stage. A significantly greater amount of *PgSPL11* was expressed in the P6 stage of the bisexual flowers compared with that in the other stages (*p* < 0.05), and its expression was lowest at P5 stage. In the P2, P7 and P8 stages of functional male flowers, the expression levels of *PgSPL11* were significantly higher than that in other stages (*p* < 0.05). The expression levels of *PgSPL12* were higher in P2 ~ P4 stages of two kinds of flowers, and showed a consistent trend. The expression levels in P5, P7 and P8 stages of amphoteric flowers were significantly lower than that in other stages (*p* < 0.05), and the expression level was the highest in P8 stage of functional male flowers. The expression levels of *PgSPL13* dropped first and then went up during the development of bisexual flowers, which showed the opposite trend in functional male flowers. *PgSPL13* showed higher expression in functional male flowers compared with bisexual flowers, reaching a maximum value in P8 stage of the bisexual flower and P6 stage of the functional male flower. The expression level of *PgSPL14* was higher in P3 stage and lower in other stages. In functional male flowers, the expression levels of *PgSPL14* were the highest in P4 and P7 stages, followed by P2 and P5 stages.

It can also be seen from Fig. 8 that the expression levels of *PgSPL2*, *PgSPL13* and *PgSPL14* in functional male flowers at P1 ~ P8 stages were higher than those in bisexual flowers. The expression levels of *PgSPL2*, *PgSPL3*, *PgSPL6*, *PgSPL11* and *PgSPL14* in young leaves were significantly higher than those in buds and stems (*p* < 0.05). The expression levels of *PgSPL5*, *PgSPL12* and *PgSPL13* in flower buds were significantly higher than that in leaves and stems (*p* < 0.05).

### Cloning and sequence analysis of *PgSPL5* and *PgSPL13*

**Fig. 9:**
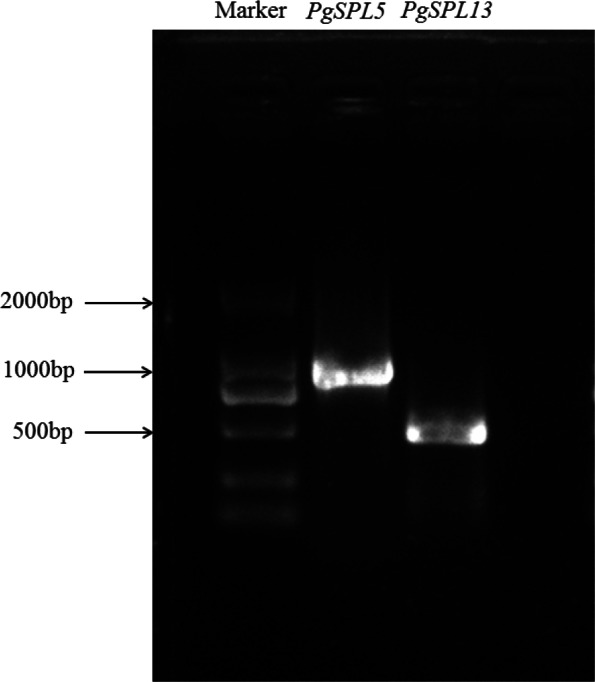
PCR amplification of *PgSPL5* and *PgSPL13* genes. Excess gel is cropped in the image

**Fig. 10:**
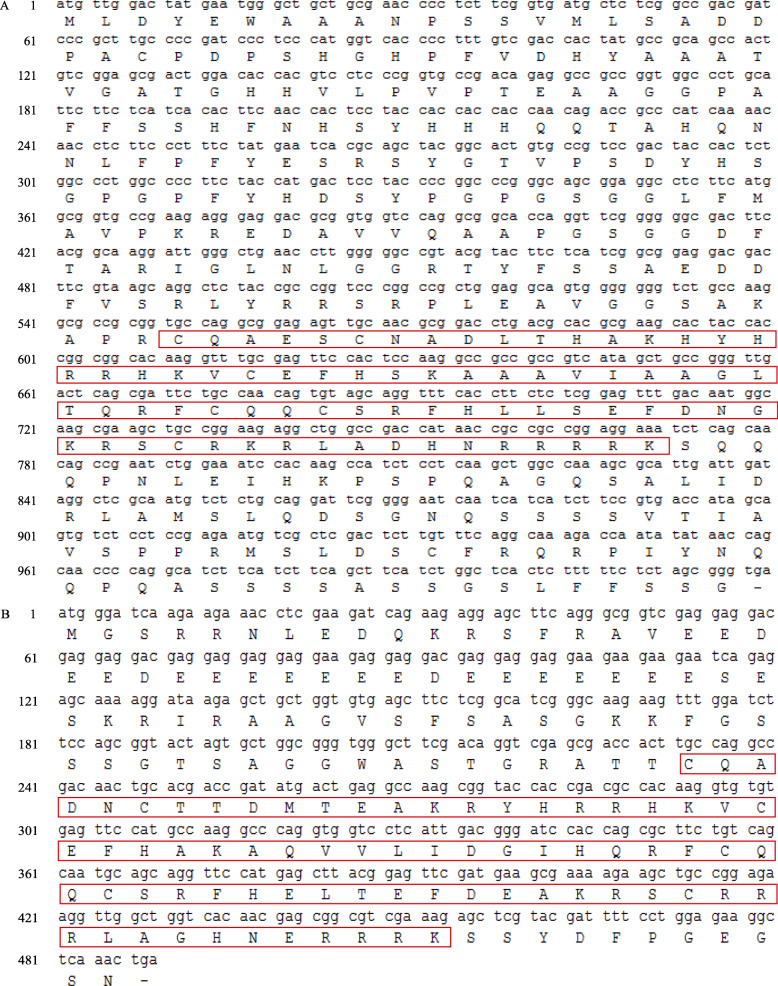
The coding sequences of *PgSPL5* (**a**) and *PgSPL13* (**b**) genes. The red box is the SBP domain

*PgSPL5* and *AtSPL8* were clustered into the same group in the phylogenetic trees, and the reconstructed phylogenetic tree also showed that *PgSPL13* was on the same branch as *AtSPL3*. Studies have shown that *AtSPL8* played an important role during anther development [36], and *AtSPL3* had a very important function in the development of *Arabidopsis* flowers [34, 35]. Therefore, we speculated that *PgSPL5* and *PgSPL13* also play roles in pomegranate flower development. Figure 8 showed that the expression levels of *PgSPL5* and *PgSPL13* in flower buds were significantly higher than those in leaves and stems (*p* < 0.05), which further indicated that these two genes have important functions in pomegranate flower development. Accordingly, we chose these two genes for cloning. Using ‘Taishanhong’ pomegranate cDNA as templates, *PgSPL5* primers and *PgSPL13* primers were used for PCR amplification respectively. The electrophoresis bands were consistent with the expected sizes of the target fragments (Fig. 9). The coding region sequences of *PgSPL5* and *PgSPL13* were obtained by sequencing, which were 1020 bp and 489 bp, encoding 339 and 162 amino acids, respectively. The molecular weight of the proteins was predicted as 36,911.79 kDa and 18,563.02 kDa, respectively. Both genes contained a complete SBP conserved domain (Fig. 10).

### Subcellular localization of *PgSPL5* and *PgSPL13*

**Fig. 11:**
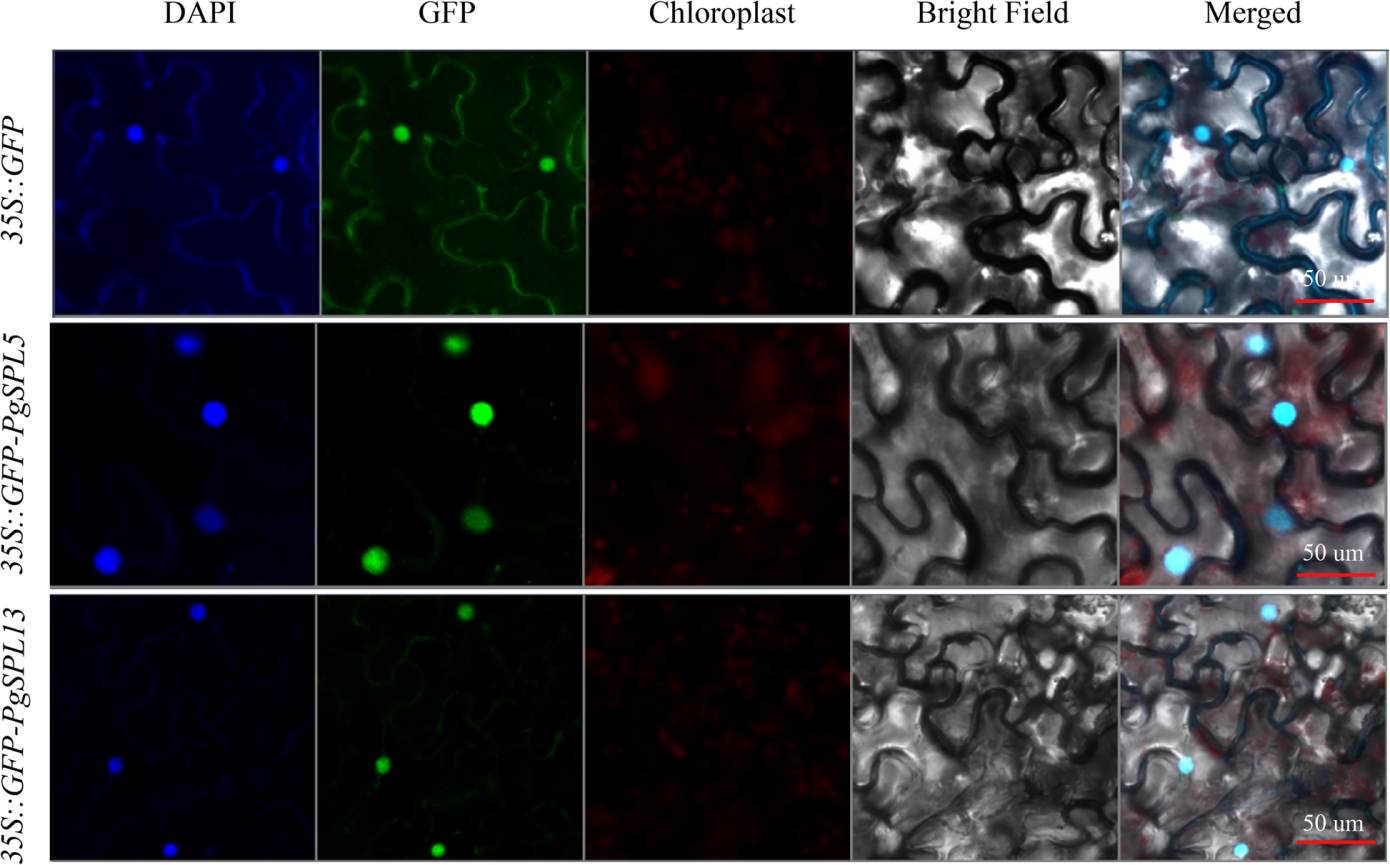
Subcellular location of PgSPL5 and PgSPL13 proteins in tobacco leaves

The subcellular localization of *PgSPL5* and *PgSPL13* was predicted by using the online tool Cell-PLoc [45], and it was found that they were both located in the nucleus. In order to verify the above predicted results, we transiently expressed 35S::GFP-PgSPL5 and 35S::GFP-PgSPL13 fusion proteins in tobacco leaves, and found that these two genes were indeed located in the nucleus (Fig. 11). It was speculated that *PgSPL5* and *PgSPL13* might be involved in the regulation of gene expression.

### Effects of exogenous hormones on expression of *PgSPL5* and *PgSPL13*

It was found that proper spraying of plant growth regulators could promote flower bud differentiation, induce female flower formation, increase plant stress resistance and increase yield. To investigate whether the expression patterns of *PgSPL5* and *PgSPL13* genes were affected by different growth regulators, the detailed expression patterns in response to different treatments were analysed by qRT-PCR and showed a great variation among different treatment groups.

**Fig. 12:**
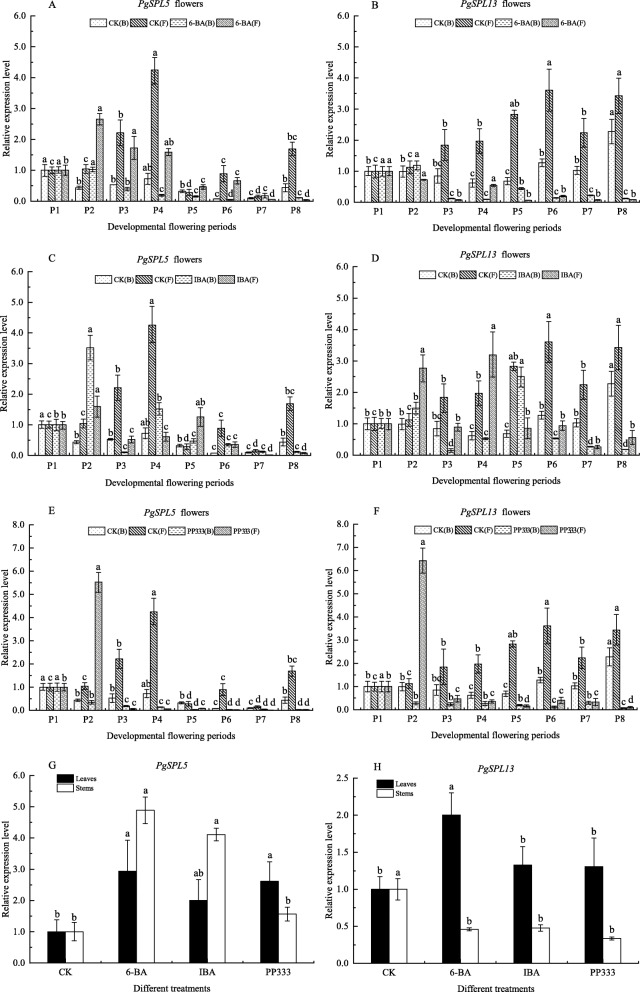
Expression levels of *PgSPL5* and *PgSPL13* following different treatments in different organizations. **a-h** are the expression levels of *PgSPL5* and *PgSPL13* in bisexual flowers, functional male flowers, leaves and stems, respectively. In the illustration (B) and (F) represent bisexual flowers and functional male flowers respectively. The vertical bar in bar graph is standard error. Bars with different letters within each panel are significant differences at *p* < 0.05 according to the Turkey’s test

As shown in Fig. 12a and b, the expression level of *PgSPL5* was significantly up-regulated at the P2 stage of the two types of flowers under 6-BA treatment, and the expression was up-regulated but not significant at the P7 stage of bisexual flowers. In other developmental stages of bisexual flowers, the expression of *PgSPL5* was down-regulated after exogenous 6-BA treatment, and *PgSPL5* expression was significantly down-regulated at the P3, P4 and P8 stages of functional male flowers. Significantly increased expression of *PgSPL5* was observed in leaves and stems under 6-BA treatment (Fig. 12g), and this change was more pronounced in stems. *PgSPL13* was up-regulated at P2 stage of bisexual flowers, and down-regulated at all other stages after 6-BA treatment. The expression of *PgSPL13* in leaves was significantly up-regulated under 6-BA treatment, while in stems it was down-regulated (Fig. 12h).

After IBA treatment (Fig. 12c, d), we found that the expression of *PgSPL5* was down-regulated at the P3 and P8 stages of bisexual flowers, and up-regulated at other developmental stages of bisexual flowers, while the up-regulation was extremely significant at the P2 stage. At P2 and P5 stages of functional male flowers, the expression levels of *PgSPL5* were significantly up-regulated, but down-regulated at other stages under IBA treatment. In leaves and stems, the expression of *PgSPL5* under IBA treatment showed the same change as 6-BA treatment, but this change was not as obvious as 6-BA treatment. *PgSPL13* was significantly up-regulated in P2 and P5 stages of bisexual flowers and P2 and P4 stages of functional male flowers, and down-regulated at other stages of flower development after IBA treatment. Similarly, the expression of *PgSPL13* presented the same changes in leaves and stems after IBA treatment as 6-BA treatment.

We can see from Fig. 12e and f, the expression of *PgSPL5* and *PgSPL13* were significantly up-regulated in P2 stage of functional male flowers after spraying exogenous PP333, while the expression of these two genes was inhibited in other stages of flower development. *PgSPL5* was up-regulated in leaves and stems, while *PgSPL13* was up-regulated in leaves but down-regulated in stems. This is consistent with the trend observed within 6-BA and IBA treatments.

## Discussion

In recent years, many studies have pointed out that the SPL transcription factor family play fundamental roles in plant growth and development and governing a variety of physiological processes [19, 46, 47]. In this study, a total of 15 *SPL* genes were identified from the whole genome of ‘Taishanhong’ pomegranate using bioinformatics methods. The information we obtained from the phylogenetic analysis was that these 15 *SPL* genes were clustered into six subgroups. This result indicated that *P. granatum*, *A. thaliana*, *M. domestica* and *Vitis vinifera* were relatively conserved during the evolution [48]. Gene structure and motif composition analyses showed that *PgSPL* genes within the same group shared similar motifs and exon-intron organization, suggesting that these genes may derived from the same ancestral gene and perform similar functions in plant growth and development [49]. Compared with other subgroups, there are many more complex conserved domains in subgroups G1 and G6, and members of these groups may have other functions [50].

The role of *cis*-acting elements influencing the gene expression is gaining emphasis in recent times [2]. This study found that all PgSPL promoters contained multiple *cis*-acting elements associated with abiotic stress or hormonal response, suggesting that PgSPL may play functions in plant response to abiotic stress and hormonal stress. In particular, the discovery of hormone-induced related elements such as auxin and gibberellin suggested that PgSPL family genes were related to the growth and development of pomegranate.

SPL family genes are regulated by miRNA156. There are 11 *SPL* genes in *Arabidopsis* that are regulated by miR156, which play multiple functions in plant growth and development [29, 30, 32]. This study predicted that in pomegranate, of the 15 SPL family members, 10 possess binding sites for miR156. In addition to the potential miR156 targets of *PgSPL2*, *PgSPL13* and *PgSPL14* in G5 subgroup were located at 3’UTR, the potential targets of other genes were located in the coding region.

Transcriptome expression data showed that most *PgSPL* genes are expressed in leaves, roots, flowers, seed coats amd pericarps, except that very few *PgSPLs* are not expressed in a mixture of pericarp and various tissues.This indicates that the PgSPL family genes are involved in pomegranate leaf development, phase transition, floral development and fruit ripening and other processes. Most *PgSPL* genes were highly expressed in differently developmental periods of bisexual flowers and functional male flowers, these data support the hypothesis that *PgSPL* genes play an important role in pomegranate flowering. At the same time, it was found that the expression of *PgSPL4* in various tissues was not significantly different, which may be related to the mutation of the C3H region of the zinc finger structure of the gene encoding protein that affected its gene function. *PgSPL8*, *PgSPL9* and *PgSPL10* are generally highly expressed in all tissues, which may be related to the existence of other more conserved motifs. Additionally, the level of *PgSPL15* was relatively low in all tissues. Therefore, it can be presumed that its role in regulating pomegranate development was weak. We also analyzed the expression differences of *miR156a-5p* and *PgSPL13* during the three different stages of pomegranate bisexual flower and functional male flower development, it was found that their expression levels showed an opposite tendency, which was consistent with the conclusion that miR156 negatively regulated the expression of its target genes [29].

Eight *PgSPLs* with significant differences in expression in different tissues of pomegranate were analyzed by qRT-PCR. The results showed that the expression levels of *PgSPL2*, *PgSPL13* and *PgSPL14* in functional male flowers were higher than those in bisexual flowers from P1 to P8 stages, showing a similar expression pattern. The expression patterns of *PgSPL6* and *PgSPL11* in the two flowers were also similar, which may be related to their clustering in the same subgroup and playing similar functions [50]. Part of flower bud pistil abortion during development of pomegranate to form functional male flowers, and functional male flower pistil abortion is due to abnormal ovule development, can not form egg cells [51–53]. Studies have shown that flower bud longitudinal diameter 5.1 ~ 15.0 mm is an important stage of pomegranate ovule development [54]. *PgSPL5* had higher expression in functional male flowers than in bisexual flowers. At P2 ~ P4 (5.1 ~ 12.0 mm) stages of functional male flowers, the expression levels of *PgSPL5* were significantly increased. Therefore, we speculated that *PgSPL5* may be involved in the regulation of functional male flower formation in pomegranate. *PgSPL12* showed similar expression patterns in the two types of flowers before ovule development abnormality, and it mainly performed functions in functional male flowers after ovule development abnormality. *PgSPL12*, *AtSPL9* and *AtSPL15* were clustered in the same branch, while *AtSPL9* and *AtSPL15* could regulate flowering time [28] and induce *Arabidopsis* flowering [30]. This indicated that *PgSPL12* also played an important role in flower development. The expression levels of *PgSPL13* were relatively stable in two types of flower development, which indicated that *PgSPL13* played a role in pomegranate flower organ development. We also found that the expression levels of *PgSPL2*, *PgSPL3*, *PgSPL6*, *PgSPL11* and *PgSPL14* in young leaves were significantly higher than those in buds and stems. This suggested that these genes played important roles in leaves development. The expression levels of *PgSPL5*, *PgSPL12* and *PgSPL13* in flower buds were significantly higher than that in leaves and stems, which further indicated that these three genes had important functions in the development of pomegranate flowers.

In *Arabidopsis*, some *AtSPL* genes such as *SPL3* and *SPL8* are involved in the response to GA signal transduction in plant development such as flowering transformation [34] and anther development [39]. Many studies have shown that *SPL3* has a very important function in the development of *Arabidopsis* flowers [35, 38, 55]. In addition, *SPL3*, *SPL4* and *SPL5* are involved in the control of flowering time and developmental transition of *Arabidopsis* [56]. Studies have found that homologs from different species usually share a similar function in the biological process. For example, the orthologous genes *OsSPL13*, *ZmSBP6* and *AmSBP1* of *SPL3* are involved in inflorescence and flower in *O. sativa* [13], *Zea mays* [57] and *A. majus* [58], respectively. In this study, we cloned *SPL8* and *SPL3* homologous genes *PgSPL5* and *PgSPL13* from ‘Taishanhong’ by homologous cloning technology, and the subcellular localization verification proved that they performed their functions in the nucleus. We studied their spatiotemporal expression characteristics under the treatment of exogenous hormones, and found that after exogenous hormone treatment, the expression of *PgSPL5* and *PgSPL13* were up-regulated or down-regulated by different plant hormones. They were differentially expressed in leaves, stems, bisexual flowers, functional male flowers and other tissues of pomegranate, and each of them respond differently to various different hormones. It indicates that these two genes may be involved in the response process of different plant hormone signal transduction in pomegranate development. We found that in flower tissues, this response was stronger when the buds were young (P2 stage), and the levels of these two genes were significantly affected by the 6-BA treatment in leaves and stems. This study only analyzed the possible functions of the two genes *PgSPL5* and *PgSPL13* in the development of pomegranate. However, how these two genes perform their functions and whether there is a mechanism between them remain to be further studied.

## Conclusions

In this study, we identified and characterized 15 *SPL* genes in pomegranate. Phylogenetic analysis indicated that the PgSPLs were divided into six subgroups, with a similar distribution of conserved motifs and exon-intron organization in the members of each subgroup. And there are 10 *SPL* genes in pomegranate that are regulated by miR156. Based on the transcriptome data of different tissues, we found certain *PgSPL* genes played significant roles in pomegranate growth and development. qRT-PCR analysis showed that *PgSPL2*, *PgSPL3*, *PgSPL6*, *PgSPL11* and *PgSPL14* played roles in leaves development of pomegranate. *PgSPL5*, *PgSPL12* and *PgSPL13* played roles in pomegranate flower development. The subcellular localization results showed that *PgSPL5* and *PgSPL13* proteins were located in the nucleus. Furthermore, exogenous plant growth regulator induction experiments showed that *PgSPL5* and *PgSPL13* were involved in the response process of different plant hormone signal transduction in pomegranate development. And we found that in flower tissues, this response was stronger when the buds were young (P2 stage), and the levels of these two genes were significantly affected by the 6-BA treatment in leaves and stems.

## Methods

### Identification and sequence analysis of SPL gene family

The HMM profile of the Pfam SBP/SPL domain (PF03110) [8] was performed against ‘Taishanhong’ pomegranate protein databases using HMMER 3.0 (http://hmmer.org/) software package (Evalue <1e^− 5^) [1], duplication was removed and SPL protein sequences were screened and selected. SPL protein sequences of *A. thaliana*, *E. grandis*, *M. domestica* and *Vitis vinifera* in PlantTFDB database (http://planttfdb.gao-lab.org/index.php) [59] were used as queries to perform BLAST against the pomegranate genome database (E-value <1e^− 5^, identity > 50%) [3]. Moreover, all obtained SPL protein sequences were further analyzed on CDD website (https://www.ncbi.nlm.nih.gov/cdd) [60], and SMART (http://smart.embl-heidelberg.de/) [61] to verify the conserved domains of SBP domain.

The online tool ExPaSy-Protparam (https://web.expasy.org/protparam/) [62] was used to predict and analyze the physical and chemical properties of pomegranate SPL family, such as molecular weight and isoelectric point. Subcellular localization was predicted using the online website (http://www.csbio.sjtu.edu.cn/bioinf/Cell-PLoc-2/) [45].

### Phylogenetic analysis

In order to explore the phylogenetic relationship of pomegranate SPL family genes, SPL proteins of *A. thaliana* (17), *M. domestica* (27) and *Vitis vinifera* (18) were downloaded for multiple sequences alignment. An unrooted Neighbor-Joining (NJ) phylogenetic tree was constructed with all of SPL protein sequences using MEGA 7.0, bootstrap = 1000 repetitions, Complete deletion, and Poisson model [63]. EvolView (https://www.evolgenius.info/evolview/) [64] online was used to beautify the phylogenetic tree.

### Gene structure analysis and motif identification

Motifs of the SPL proteins were identified using MEME online tools (http://meme-suite.org/) [65] with default parameter, and the motif characteristics of the SPL proteins were obtained. The exon-intron structures of the *SPL* genes were determined based on the annotation information of ‘Taishanhong’ genome, then the results were displayed using GSDS 2.0 (http://gsds.cbi.pku.edu.cn) online tools [66].

### *Cis*-acting elements

The 1500 bp upstream sequence of the initial codon were obtained from pomegranate genome sequence for *cis*-acting element analysis by the PlantCARE (http://bioinformatics.psb.ugent.be/webtools/plantcare/html/) [67].

### Prediction of *PgSPL* genes targeted by miR156

Using psRNATarget online software (http://plantgrn.noble.org/psRNATarget/) [68] to analyze the nucleotide sequence of pomegranate SPL genes and predict the target gene site of pomegranate miR156. DNAMAN software was used to perform multiple sequence alignment between the reverse complement of PgmiR156 and PgSPL sequences [43].

### Expression patterns by transcriptome data

To examine expression patterns of *SPL* genes in different pomegranate tissues and organs, the published transcriptome data were download for expression analysis. The transcript data of bisexual flowers, functional male flowers, leaf, root, outer seed coat, inner seed coat, and pericarp of pomegranate were obtained from NCBI database (http://www.ncbi.nlm.nih.gov/) [3]. The registration numbers are ‘Dabenzi’ SRR5279388, SRR5279391, SRR5279394-SRR5279397; ‘Tunisia’ SRR5446592, SRR5446595, SRR5446598, SRR5446601, SRR5446604, SRR5446607 and SRR5678820; ‘Baiyushizi’ SRR5678819; ‘Black127’ SRR1054190; ‘nana’ SRR1055290 and ‘Wonderful’ SRR080723. Firstly, all RNA-Seq data were qualitatively controlled by fastp to obtain cleaned reads. Then the sequence was indexed with pomegranate transcriptome data. Through Kallisto 0.44.0 software [69], and the gene expression was further calculated and analyzed. The corresponding expression level (TPM value) of *SPL* genes was thus obtained. TPM value was transformed into Log_2_(TPM + 1). Finally, the heat map of *PgSPL* was drawn with TBtools [1].

At the same time, the expression of miR156 of pomegranate bisexual flowers and functional male flowers at three different stages (bud vertical diameter of buds were 3.0 ~ 5.0 mm, 5.1 ~ 13.0 mm, 13.0 ~ 20.0 mm) was analyzed.

### Plant material and exogenous hormone treatments

Research conducted at Baima Base for Teaching and Scientific Research of Nanjing Forestry University. The tested cultivars were 6-year-old ‘Taishanhong’ pomegranate. Three plant growth regulators were selected and the spraying concentration was as follows (Table 2) [70]. Each group consisted of 3 plants, and each was sprayed to drip. Individual plants were treated on October 15, 2019 (dormancy period), April 15, 2020 (spring leaf expansion period), May 10 (initial flowering to full flowering period) and May 25 (end of full flowering period), respectively.

**Table 2:** Treatment types and concentrations

Types of treatment	Concentrations (mg/L)
6-BA	100
IBA	20
PP333	1000
CK	Water

*6-BA* 6-Benzylaminopurine, *IBA* Indole-3-butyric acid, *PP333* Paclobutrazol

**Fig. 13:**
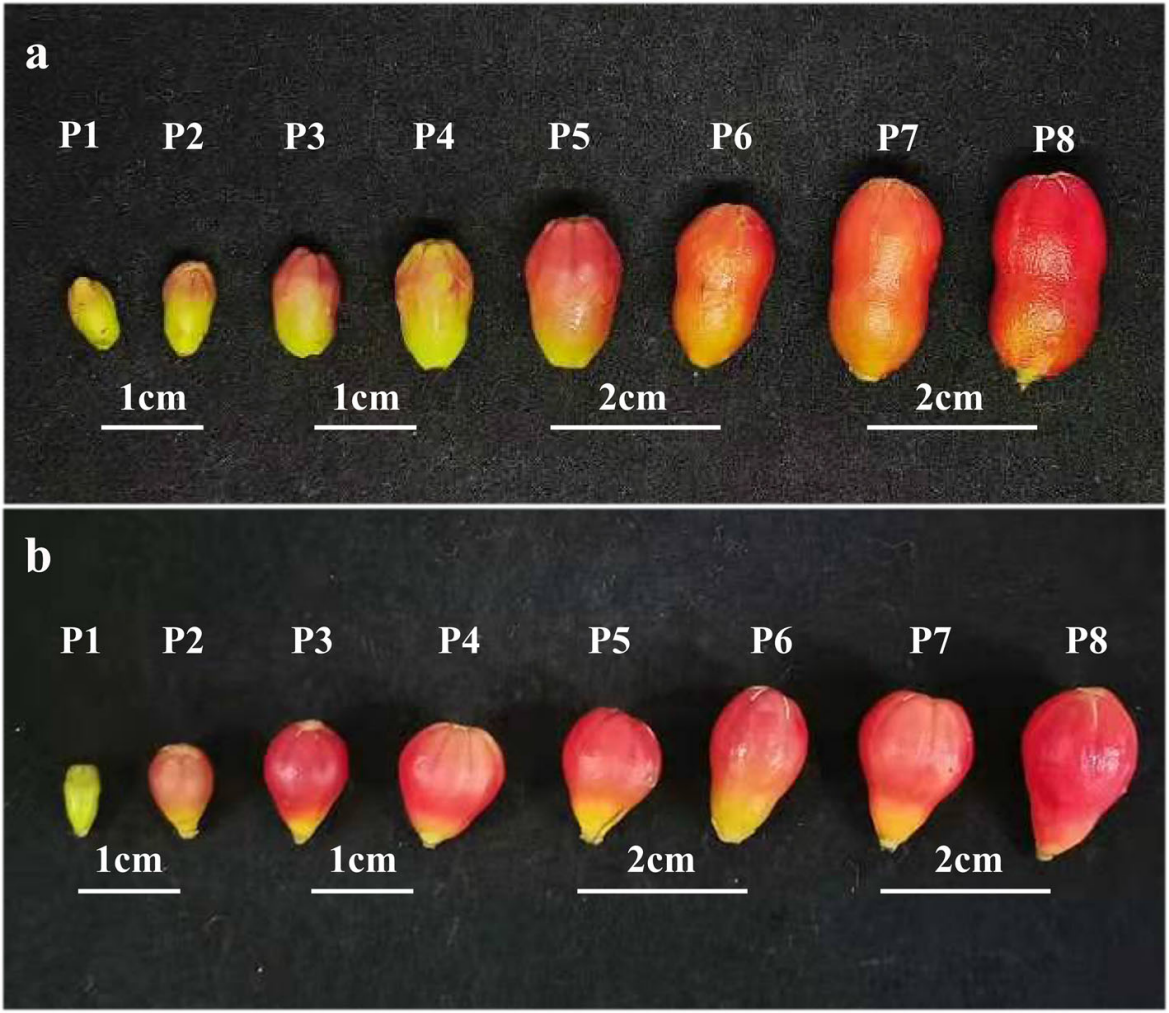
Bisexual flower (**a**) and Functional male flower (**b**) at different developmental stages (CK)

No permission is required for sample collection. New leaves and stems were collected after 7 days of each treatment from May 10, and the bisexual flowers and functional male flowers at different developmental stages of pomegranate were collected. Referring to Chen and Zhao’s study on the morphological and embryological differences of pomegranate flowers [3, 71], the flower buds were divided into eight stages according to their longitudinal diameter (Fig. 13): 3.0 ~ 5.0 mm (P1), 5.1 ~ 8.0 mm (P2), 8.1 ~ 10.0 mm (P3), 10.1 ~ 12.0 mm (P4), 12.1 ~ 14.0 mm (P5), 14.1 ~ 16.0 mm (P6), 16.1 ~ 18.0 mm (P7), and 18.1 ~ 20.0 mm (P8). Samples were frozen with liquid nitrogen and stored in − 78 °C refrigerator for reserve.

### RNA isolation and gene cloning

Total RNA was extracted using the BioTeke plant total RNA extraction kit (BioTeke Corporation, Beijing, China), and first-strand cDNA was prepared using a reverse transcription kit-PrimeScript™ RT reagent Kit with gDNA Eraser (TaKaRa Bio Tech Co., Ltd., Beijing, China). Then the cDNA was used as template for gene clone and expression analysis on SPL family genes at different developmental stages of pomegranate. According to DNA and CDS sequences, specific quantitative primers (Table 3) were designed using Oligo 7 software [72].

**Table 3:** Primers used in this study

Primer	Primer sequence(5′-3′)	Annotation
*PgSPL5*	F: ATGTTGGACTATGAATGGGC R: TCACCCGCTAGAGAAAAAGAG	Gene clone
*PgSPL13*	F: ATGGGATCAAGAAGAAACCTCGAAG R: TCAGTTTGAGCCTTCTCCAGGAAAA	Gene clone
GFP*-PgSPL5*	F:gagaacacgggggactctagaATGTTGGACTATGAATGGGC R:gcccttgctcaccatggatccTCACCCGCTAGAGAAAAAGAG	subcellular localization
GFP*-PgSPL13*	F:gagaacacgggggactctagaATGGGATCAAGAAGAAACCTCGAAG R:gcccttgctcaccatggatccTCAGTTTGAGCCTTCTCCAGGAAAA	subcellular localization
qRT-*PgSPL2*	F:AAGAGAGACTTCACCACCGAG R: GTGAAACCTGCTGCATTGCT	Gene expression
qRT-*PgSPL3*	F: AACAGCCGAGACTCATCCC R: GAGGTCCTTGTTACACCCGTA	Gene expression
qRT-*PgSPL5*	F: GAAATCTCAGCAACAGCCGAAT R: CTTTGCCTGAAACAAGAGTCG	Gene expression
qRT-*PgSPL6*	F: AATGCAGCAGGTTTCATTCCC R: TCGTTTTCCGACTTGACAGC	Gene expression
qRT-*PgSPL11*	F: TTCCCAGCCATTCCATCCTCG R: GGCTTCGCTATACCAAGTCCT	Gene expression
qRT-*PgSPL12*	F: AAGTCCCCTAAAGTCATCGTT R: TGAAGTCCATCACAAAGCCT	Gene expression
qRT-*PgSPL13*	F: CGACAACTGCACGACCGAT R: CGGCAGCTTCTTTTCGCTTC	Gene expression
qRT-*PgSPL14*	F: TCGACGATACCAAGAGAAGCTG R: AATTCTCCCCCGATCATCGAC	Gene expression
*PgActin*	F: AGTCCTCTTCCAGCCATCTC R: CACTGAGCACAATGTTTCCA	Gene expression

Each 50 μL of reaction mixture contained 25 μl 2× Taq Plus Master Mix, 1 μl each of the upstream/downstream primers, 2 μl of cDNA template and 21 μl Nuclease-free ddH2O. PCR program was set as follows: pre-denaturation at 95 °C for 3 min; 95 °C for 15 s, 58 °C for 45 s, 72 °C for 1 min, a total of 35 cycles; 72 °C for 5 min. Firstly, the PCR products were separated by 1% agarose gel electrophoresis, and the target fragment was recovered by gel cutting. The recombinant plasmid was connected to the overexpression vector, and transformed into competent *E. coli* DH5α. Then, the positive monoclonal detected by PCR was selected and sent to Shanghai Bioengineering Company for sequence. Finally, the sequences obtained by sequencing were translated into amino acid sequences using ExPaSy-Translate online tool (https://web.expasy.org/translate/) [62].

### Subcellular location analyses

To verify the location of *PgSPL5* and *PgSPL13*, the full-length coding sequence (CDS) without stop codon was cloned into the pBI121 and C-terminal fused with the green fluorescent protein (GFP). Primers for the restriction sites at both ends of the target gene were designed (Table 3). The product was recovered from the recombination ligation gel, and the recombinant plasmid obtained from the ligation was introduced into Agrobacterium GV3101 by freeze-thaw method. The vector GV3101 (*A. tumefaciens*) harbouring 35S::GFP-PgSPL and control vector were infiltrated into tobacco (*Nicotiana benthamiana*) leaves. After 36 ~ 48 h, Fluorescence images were captured using an LSM 710confocal laser-scanning microscope (Zeiss, Jena, Germany) [72, 73].

### Real-time fluorescent quantitative PCR analysis (qRT-PCR)

The expression of *PgSPLs* in different tissues was analyzed by qRT-PCR, and the expression of *PgSPL5* and *PgSPL13* under different growth regulators was analyzed. Specific quantitative primers (Table 3) were designed, and pomegranate *PgActin* gene was used as an internal reference. Each 20 μL of reaction mixture contained 10 μL of TB Green Premix Ex Taq, 0.4 μL of ROX Reference Dye II, 0.4 μL of upstream/downstream primers, 2 μL of cDNA template and 6.8 μL of ddH2O. qRT-PCR was performed using a 7500 fast Real-Time PCR system (Applied Biosystems, CA, USA) with three biological and three technical replicates for each cDNA sample. The thermal cycler was set as follows: pre-denaturation at 95 °C for 30 s, 40 cycles of 95 °C for 5 s and 60 °C for 34 s; the dissociation stage was set as follows: 95 °C for 15 s, 60 °C for 60s and 95 °C for 15 s. The data was quantitatively analyzed by the 2^-ΔΔCt^ method [74].

### Data analysis

All data of qRT-PCR were analyzed with one-way ANOVA, and multiple comparisons were evaluated with the Turkey’s test (*p* < 0.05) using the SPSS program (Version 19.0. Chicago, IL, USA) based on the values of three complete randomized blocks.

## Supplementary Information

**Additional file 1.**

## Data Availability

The whole genome data of pomegranate is downloaded from the NCBI database (https://www.ncbi.nlm.nih.gov/search/all/?term=ASM220158v1), and the accession number is ASM220158v1. The transcriptome data is obtained from NCBI (https://www.ncbi.nlm.nih.gov/Traces/study/), and the accession numbers are SRP103147 and SRP100581. The SPL protein sequences of *A. thaliana*, *E. grandis*, *M. domestica* and *Vitis vinifera* are downloaded from the PlantTFDB database (http://planttfdb.gao-lab.org/index.php). Public access to all databases is open. The datasets supporting the conclusions of this article are included within the article (and its additional files).

## Abbreviations

SPL: SQUAMOSA promoter-binding protein-like
*A. thaliana*: *Arabidopsis thaliana*
*E. grandis*: *Eucalyptus grandis*
IBA: Indole-3-butyric acid
6-BA: 6-Benzylaminopurine
PP333: Paclobutrazol
RNA: Ribonucleic Acid
cDNA: complementary DNA
qRT-PCR: Quantitative real-time polymerase chain reaction
