# Supplementary figures and images for: Genome-wide identification, gene cloning, subcellular location and expression analysis of SPL gene family in P. granatum L
Source: BMC Plant Biol. 2021 Aug 28;21:400. doi: 10.1186/s12870-021-03171-7 (PMC8399725; doi:10.1186/s12870-021-03171-7)

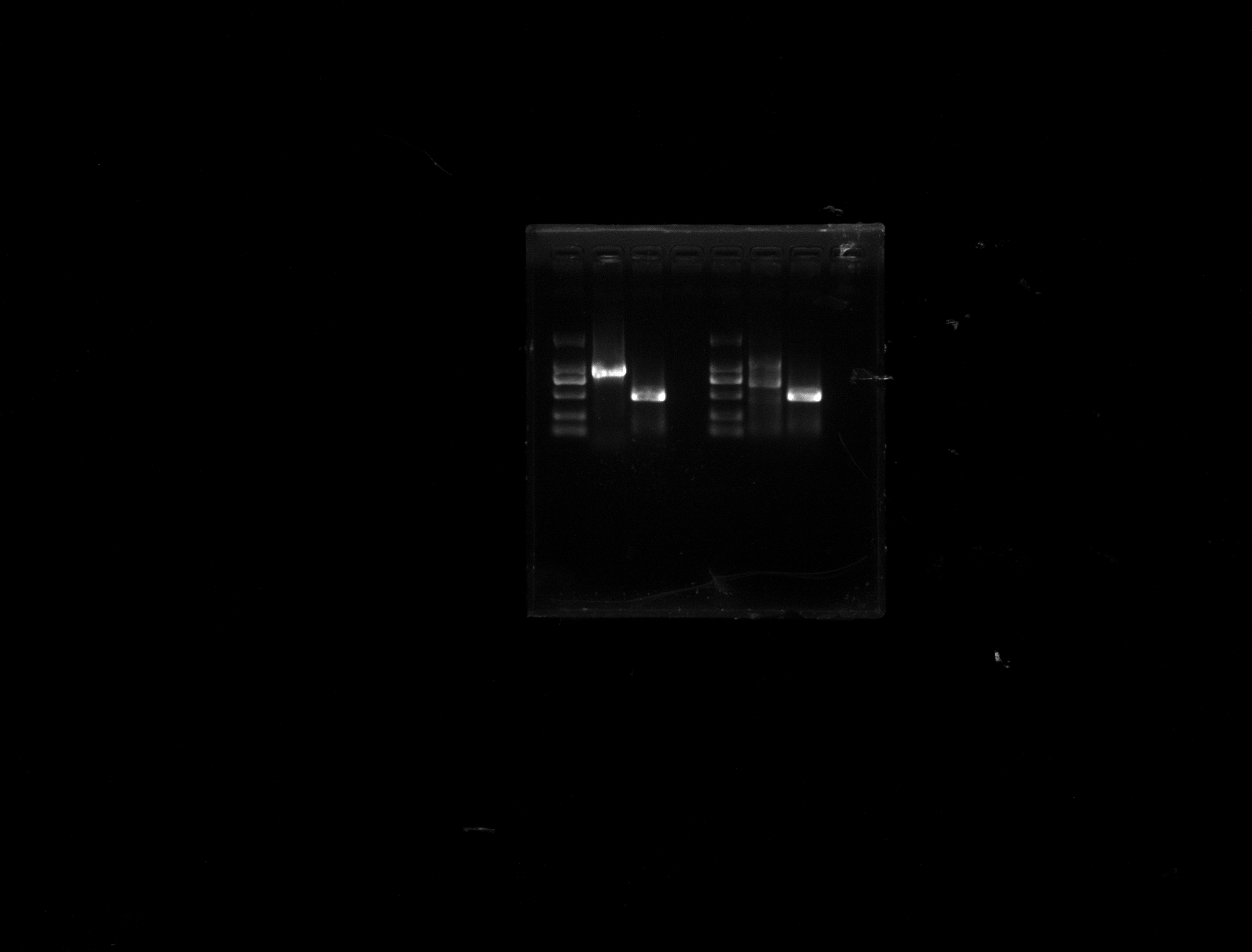

Supplement: Supplementary file 1 — Additional file 1. [file 12870_2021_3171_MOESM1_ESM.bmp]
